# Occupational COPD—The most under‐recognized occupational lung disease?

**DOI:** 10.1111/resp.14272

**Published:** 2022-05-05

**Authors:** Nicola Murgia, Angela Gambelunghe

**Affiliations:** ^1^ Section of Occupational Medicine, Respiratory Diseases and Toxicology University of Perugia Perugia Italy

**Keywords:** airways, bronchitis, COPD, emphysema, occupational, VGDF, workers

## Abstract

Chronic obstructive pulmonary disease (COPD) is caused by exposure to noxious particles and gases. Smoking is the main risk factor, but other factors are also associated with COPD. Occupational exposure to vapours, gases, dusts and fumes contributes to the development and progression of COPD, accounting for a population attributable fraction of 14%. Workplace pollutants, in particular inorganic dust, can initiate airway damage and inflammation, which are the hallmarks of COPD pathogenesis. Occupational COPD is still underdiagnosed, mainly due to the challenges of assessing the occupational component of the disease in clinical settings, especially if other risk factors are present. There is a need for specific education and training for clinicians, and research with a focus on evaluating the role of occupational exposure in causing COPD. Early diagnosis and identification of occupational causes is very important to prevent further decline in lung function and to reduce the health and socio‐economic burden of COPD. Establishing details of the occupational history by general practitioners or respiratory physicians could help to define the occupational burden of COPD for individual patients, providing the first useful interventions (smoking cessation, best therapeutic management, etc.). Once patients are diagnosed with occupational COPD, there is a wide international variation in access to specialist occupational medicine and public health services, along with limitations in workplace and income support. Therefore, a strong collaboration between primary care physicians, respiratory physicians and occupational medicine specialists is desirable to help manage COPD patients' health and social issues.

## Graphical abstract

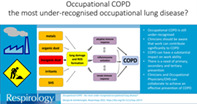

**See related** Editorial

## INTRODUCTION

Chronic obstructive pulmonary disease (COPD) is a common disease defined by chronic respiratory symptoms and airflow limitation.^1^ Respiratory symptoms usually describe the major clinical COPD phenotypes: emphysema, characterized mainly by exertional dyspnoea, and chronic bronchitis, where chronic cough with phlegm is the predominant symptom. The two clinical phenotypes can be present simultaneously in the same COPD patient, manifesting in a mixed phenotype. In COPD, airway obstruction is typically non‐reversible or not fully reversible.^1^

According to the 2017 Global Burden of Disease (GBD) study, COPD is the third leading cause of death worldwide, accounting for at least 3 million deaths every year.^2^

The global prevalence of COPD is high, at almost 4% of the population.^2^ Prevalence increases up to 10% among people over 40 years of age.^3^ The prevalence of COPD is underestimated and varies across countries, reflecting the specific age distribution of the population, healthcare resourcing, variable exposure to risk factors and the prevalence of predisposing genetic conditions.

COPD also causes significant socio‐economic burden, accounting for more than 3% of all healthcare expenditure.^4^ In the United States, the direct cost of COPD is more than 30 million dollars yearly, while indirect costs are 20 million dollars per year.^5^ COPD is also one of the most important causes of disability with a very high rate of years of life lost and years lived with disability.^6^

The Global Initiative for Obstructive Lung Disease (GOLD) defines COPD as a preventable disease caused by significant exposure to particles and noxious gases and influenced by host factors, highlighting the importance of exposure to exogenous risk factors and the presence of predisposing factors, such as genetic background, lung growth/development and pre‐existing asthma.^1^

The most important risk factor is tobacco smoking. After the first reports indicating tobacco smoking as a risk factor for COPD, more than 12,000 papers have been published on this subject. In the United States during the 1980s, tobacco smoke was the leading cause of 80%–90% cases of COPD.^7^ In population‐based studies, the population attributable fraction (PAF) of COPD due to smoking could reach 97.6%, ranging between 39.6% and 76.2% in longitudinal studies.^8^

Other important risk factors for COPD are occupational and environmental exposures, respiratory infections, lower socio‐economic status, dietary habits^1^ and early life events which influence lung function trajectories.^9^

## HISTORICAL CONTEXT

The current definition of COPD is rather recent (20th century), but its clinical counterparts, chronic bronchitis and emphysema, were widely known long before the pathogenesis was understood.

During the 19th century, some authors reported a high prevalence of chronic bronchitis and similar diseases in subjects working in ‘dusty trades’, especially those involved in Series organic dust.^10^, ^11^ Analogous, but more in‐depth, reports were made in the mid‐20th century, involving miners and other dust‐exposed trades.^12^, ^13^ In the same period, tobacco smoking was clearly identified as the leading risk factor for chronic bronchitis, emphysema and non‐reversible airway obstruction, catalysing the interest of researchers and clinicians.

It was not until the 1970s and 1980s that there were new reports on the association between occupational exposures and airflow limitation or chronic bronchitis, coming mainly from cohort studies among miners and workers exposed to cotton dust.^14^ Moreover, in this period, the first papers appeared on the association between cadmium exposure and emphysema.^15^ In the late 1980s, the first significant reviews on the association between chronic airflow limitation and occupational exposures were published.^16^, ^17^ Those papers awakened a new interest in this field leading to cohort studies, case–control studies and population‐based studies to elucidate the association between occupation and COPD. This body of evidence stimulated the publication of a comprehensive statement by the American Thoracic Society (ATS) and the European Respiratory Society (ERS) in 2003.^18^ This statement was recently updated in 2019, confirming the occupational burden of COPD due to gas, dust, vapours and fumes exposure.^19^

## OCCUPATIONAL COPD UNDERDIAGNOSIS

COPD is already an underdiagnosed disease. Recent studies suggest that nearly 70% of persistent airflow obstruction cases are not diagnosed because of spirometry underuse^20^ and a lack of awareness in some specific groups (young people, current or never smokers and those with mild symptoms).^21^ Despite the amount of data supporting the role of occupational exposure on the occurrence of COPD, the diagnosis of occupational COPD is more rare than would be expected.

In Italy, reporting of occupational diseases to the Italian National Institute for Insurance Against Accidents at Work (INAIL) is compulsory for every physician; however, only 575 cases of occupational COPD were reported to INAIL in 2019.^22^ Based on incidence data,^23^ numbers at least 10 times higher are expected. Similar concerns are present in other countries.^24^

The reasons for underdiagnosis of occupational COPD are manifold. In some countries, work‐related COPD in smokers does not result in workers' compensation, leaving the definition of ‘compensated occupational COPD’ only for cases in which other strong risk factors such as smoking are not present. Of the 575 cases of possible occupational COPD reported in Italy in 2019, only 55 were compensated.^22^ This policy may discourage physicians from reporting COPD for the purpose of workers' compensation insurance. Furthermore, interest and medical education on work‐related risk factors of COPD is recent and inconsistent; hence, some physicians do not fully consider the workplace a possible cause of the disease. Training in occupational medicine is often considered inadequate by general practitioners, who admitted a lack of occupational history taking as the main reason behind the underdiagnosis of occupational diseases.^25^ Moreover, some patients who are still employed may not report a particular workplace exposure for fear of possible consequences to their employment, similar to current smokers who do not want to quit smoking.^21^

Occupational COPD underdiagnosis may have significant consequences, not only on workers' compensation but also on disease progression and especially lung function decline.^26^, ^27^

For these reasons, it is crucial for healthcare professionals to ask about this specific association and consider options to prevent and reduce the occupational burden of COPD at all levels.

## THE ROLE OF OCCUPATIONAL EXPOSURES IN COPD AETIOPATHOGENESIS

### Epidemiology

Since the early studies on chronic bronchitis and dusty trades, scientific evidence on the association of workplace exposure and COPD (defined by airflow limitation) has accumulated in the last 40 years. Epidemiological evidence is based on case–control and population‐based studies, as well as on traditional industrial cohort studies. Some cohort studies are prospective, allowing a more robust analysis of the role of workplace exposures in causing COPD (Box 1).

#### Cohort studies

Cohort studies are usually considered more informative than case–control studies for exploring associations between a risk factor and a disease, because of a better exposure definition; on the other hand, they are less exploratory than case–control studies because there is usually a focus on only one or few exposures. Cohort studies have been extensively applied in COPD aetiological research, suggesting a causative role of occupational exposures. Inorganic dust, organic dust and other agents were addressed as possible risk factors for COPD.

The first seminal studies^17^, ^28^ were focused on inorganic dust exposure, with industrial cohorts suggesting a role for coal dust exposure.^29^

There is a large body of scientific evidence based on coal miners' studies, characterized by an exposure–response relationship among coal dust exposure and decreased lung function, regardless of smoking habits and age^30^, ^31^, ^32^, ^33^, ^34^, ^35^, ^36^, ^37^, ^38^, ^39^, ^40^ and remaining even after a substantial reduction in the exposure.^39^, ^41^ In the United States, data from the National Institute of Occupational Safety and Health (NIOSH) coal worker health surveillance programme showed a prevalence of airway obstruction in never‐smokers of 7.7%, 16.4% among those with coal workers' pneumoconiosis and 32.3% in those with progressive massive fibrosis. The latter two categories were expected to be more exposed in terms of airborne concentration and exposure duration.^37^ Similar findings were also reported in China.^40^ One study indicates that if 100 coal miners were exposed for 35 years at 2 mg/m^3^ of respirable dust, eight of them were expected to develop a clinically important loss of forced expiratory volume in 1 s attributable to dust.^28^

Another important risk factor for chronic airway obstruction is silica dust, which could cause airflow limitation, even without silicosis.^28^, ^42^ Cohort studies of South African gold miners, Canadian hard rock miners, Western Australian miners, US molybdenum miners, German uranium miners, Welsh slate miners and Brazilian semi‐precious stone mine workers^43^, ^44^, ^45^, ^46^, ^47^, ^48^ found airflow limitation. Similar results came from studies in silica‐exposed workers in non‐mining sectors such as granite crushers, tunnel workers, construction workers, brick‐manufacturing workers, slate workers, stone carvers and grinders, ceramic workers, refractory ceramic fibres workers, moulders and coremakers Series furan resin, the silicon carbide industry, iron foundry and smelter workers.^49^, ^50^, ^51^, ^52^, ^53^, ^54^, ^55^, ^56^, ^57^, ^58^, ^59^, ^60^, ^61^, ^62^, ^63^, ^64^, ^65^

Other types of inorganic dust that may be risk factors for COPD are asbestos and cement. The typical lung function pattern of asbestosis is restrictive; however, an obstructive lung function impairment has been found in asbestos‐exposed workers in Canada.^66^, ^67^ There are some old cohort studies on cement workers reporting a reduced lung function in those exposed to this type of inorganic dust.^68^, ^69^, ^70^

Recent studies in farmers have associated lifelong exposure to organic dust with the occurrence of COPD.^71^, ^72^ Exposure to other types of organic dust in the textile sector^73^ or in paper mills^74^ has been associated with COPD or accelerated lung function decline.

Other chemicals that could play a role in COPD pathogenesis are welding fumes, cadmium and irritants. Studies on welding fumes are not completely concordant. In shipyard welders and burners, a dose–exposure relationship was found between exposure to welding fumes and airflow limitation.^75^, ^76^, ^77^, ^78^ A systematic review on the effect of welding fumes on lung function found that the decline was larger in welders than in non‐welders, but it was not that large overall and may be influenced by smoking habits.^79^ Cadmium is known to cause emphysema and has also been associated with a non‐physiological lung function decline.^80^

Exposure to irritant gases has an acute effect on lung function and probably also a chronic effect. Nitrous fumes,^81^ sulphur dioxide and chlorine^82^ have been associated with lung function decline. Recently, an increased incidence of doctor‐diagnosed COPD was found in nurses, associated with the use of irritant cleaning agents.^83^

Second‐hand smoke (SHS) is a potential risk factor for COPD.^8^ Cohort studies on the association between SHS exposure at the workplace and COPD are lacking. Large population‐based studies found that workplace exposure to SHS can be a risk factor for COPD, with a PAF of 9%.^84^

Cohort studies have identified important roles for exposure to coal dust and silica dust in causing COPD. For other risk factors, the evidence is still not completely convincing, but it is enough to recommend reducing exposure based on the precautionary principle.

#### Population‐based studies

Population‐based studies allow estimation of the PAF of a disease related to a specific risk factor. This is calculated considering the extent of risk estimates and the prevalence of exposure in the population, measuring the proportion of a disease caused by that specific risk factor.

Exposure indicators can be numerous, but in the last 20 years, a broad category of ‘vapours, gases, dusts and fumes’ (VGDF) has been used extensively in population‐based studies and case–control studies to describe potential ‘at‐risk’ exposure. Information on VGDF exposure was usually collected by questionnaires as self‐reported or by specific job‐exposure matrices extrapolated from the job history of the participant.^85^ This approach tends to make the occupational exposure category more homogenous and can be useful especially when occupational exposure is not easy to identify retrospectively, as in large case–control and population‐based studies. Details on the exposure are better studied among occupational cohorts, where the occupational exposure of interest is generally clearly known from the beginning of the study period.

As mentioned before, the ATS in collaboration with the ERS produced the first statement in 2003 on occupational burden of airway diseases, considering all the studies on this topic until 2001. The statement found five papers focused on COPD as airway limitation in population‐based studies.^18^ In these five papers, the PAF due to occupational exposure to gases, dusts and fumes was a median value of 19%, ranging between 9% and 56%.

Almost 20 years later, the ATS and ERS released an update of their first statement.^19^ In this case, a higher number of studies (26) were considered in the time range 2001–2017, reflecting the better disease definition and increased interest on this topic in the last 20 years. The pooled PAF due to workplace exposure was 14%, not particularly lower than the previous estimate, despite the larger number of included studies and the expected improvement in workplace hygiene in the last 30 years.

It is important to note that papers included in the first statement were only from the United States, Europe and New Zealand, whereas the studies included in the second statement were also from other countries (China, India, Philippines and Nigeria), making this observation more globally representative. Interestingly, when only non‐smokers were considered (in six studies), the work‐related PAF increased to 31%.

Since 2017, other population‐based studies were published on this topic. A follow‐up of the European Community Respiratory Health Survey found a PAF for COPD due to occupational exposures to VGDF of 14.1%.^86^ Analysis of the 2016 GBD Study on COPD mortality indicated a PAF due to particulate matter, gases and fumes and SHS of 15.6%.^87^

In more recent years, studies performed in the United States and Europe found lower risk estimates: in the analysis of US National data, the VGDF‐related COPD PAF was between 7.4% and 10.7%^88^; in the UK, the Biobank study gave a VGDF‐related PAF of 4.8%^89^; and in the Swedish general population, PAF ranged from 3.9% and 4.7%, depending on airway obstruction definition.^90^ Another Swedish study on a different sample of the general population confirmed a PAF of 5.9% in those exposed to dust.^91^ These latter results are somewhat expected and may reflect an improvement in workplace hygiene or a ‘move’ of risk factors from the United States, Europe and Australia to other countries, from where more data on this association would be expected in the future, if strong preventive interventions are not implemented. However, the burden remains rather high in the United States and Europe, considering the prevalence of COPD and thus the large number of preventable cases related to occupational exposures. Furthermore, given the reduction of smoking rates in some countries,^92^ this trend may be surprising, but smoking and occupational exposure are interrelated factors in COPD causation. In a North American study, the risk of COPD was 1.4 for occupational exposure to VGDF alone, 2.8 for smoking alone and 6.2 in those who were exposed at work and smokers.^93^ In another study from Northern Europe, the risk of COPD associated with occupational exposure to VGDF was present only in smokers.^90^ In other studies, this possible synergistic effect was not seen, suggesting an additive effect.^94^, ^95^ The reason for this interaction is still unclear, but some authors speculate that toxic agents in cigarette smoke could be adsorbed on the particulate matter of VGDF.^96^

In conclusion, epidemiological evidence on the association between occupational exposures and COPD is rather strong for some defined exposures, such as inorganic dust. Furthermore, there is emerging evidence that many other exposures to VGDF could play a role in the aetiopathogenesis of COPD, yielding a significant burden of this disease.

### COPD and work: Is there a biological mechanism?

COPD is a complex disease where exposure to noxious particles/gases, the respiratory innate and adaptive immune response and interaction with genetic predisposing factors play the most relevant roles in pathogenesis. Noxious particles and gases, most notably tobacco smoke, can cause direct damage to the airways by reactive oxygen species (ROS). This damage and the direct interaction between gases and particles with airway epithelium are responsible for the first immunological response, which is mainly innate, involving Toll‐like receptors (TLRs), especially TLR4,^97^ the expression of pro‐inflammatory chemokines and cytokines (e.g., TNF‐α, CXCL8) and activation of the NLP3 inflammasome.^98^ This first phase is characterized by activity of macrophages and neutrophils, involved in clearing airways of noxious agents by the production and release of proteases, ROS and metalloproteinases (MMP), which may cause airway and lung damage. Macrophages also act as antigen‐presenting cells for adaptive immune activation, which is mainly characterized by a Th1 response, with CD8+ and Tc1 as leading types of cells, producing perforins and enhancing lung damage.

Also, Th17 lymphocytes and innate lymphoid cells type 3 (ILC3) may play a role in COPD, promoting neutrophil activation and chemotaxis.^99^ These factors interact with a genetic background, of which α1‐antitrypsin deficiency (AATD) is the best known, but also genes involved in MMP expression (especially MMP‐12) and other genes such as those encoding for HHIP (hedgehog interactive protein), strongly associated with lung function and COPD.^99^ Recently, important epigenetic mechanisms have been implicated in the pathogenesis of COPD, in particular the up‐ or downregulation of micro‐RNA (miRNA).^100^

This general scenario can also be applied for occupational noxious gases and particles, especially inorganic dust. After deposition along the airways, inorganic dust can interact with bronchial epithelial cells and macrophages,^101^, ^102^ causing cell damage, ROS production and release of cytokines, chemokines and proteases.^103^, ^104^ There is some evidence from studies in animals that welding fumes could produce oxidative stress and thus ROS.^105^, ^106^ In humans, controlled exposure to welding fumes containing zinc and copper caused the production of systemic inflammatory markers.^107^ Cadmium exposure, know historically to induce emphysema, can activate MMP pathway and inflammation.^108^ Exposure to organic dust could induce inflammation through the activation of ICAM‐1.^109^ Endotoxin and beta‐glucans, usually present in organic dust, may have a role in maintaining chronic inflammation.^110^

From the genetic point of view, COPD susceptibility to VGDF could be enhanced by AATD,^111^ heme oxygenase 1 polymorphism^112^ and DNA methylation.^113^

**FIGURE 1:**
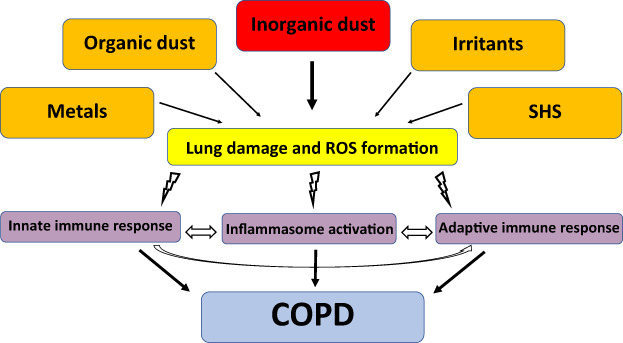
The role of occupational exposures in causing chronic obstructive pulmonary disease

In conclusion, experimental data confirm the role of occupational exposure in the pathogenesis of COPD, especially for inorganic dust, with data emerging on welding fumes, metals and irritants. A comprehensive model on the current knowledge on occupation–COPD interaction is displayed in Figure 1.

**BOX 1:** Occupational COPD aetiopathogenesis

Epidemiological studies highlight the role of occupational exposure in contributing to chronic obstructive pulmonary disease (COPD) causation Population‐based studies estimates an occupational burden of COPD of 14% Cohort studies reported a significant role of occupational exposure to inorganic dust and suggested a role for organic dust, metals and irritants Experimental studies are supporting the biological plausibility of the association between occupational exposure and COPD occurrence

## WHAT SHOULD A CLINICIAN KNOW ABOUT OCCUPATIONAL COPD?

COPD is a preventable and avoidable disease. This is the first concept that a clinician must include in her/his basic knowledge. For smoking this concept is quite clear, but not always for other emerging risk factors such as workplace exposures, environmental pollution and lung ageing. Considering occupational exposure, data from three large population‐based studies (Burden of Obstructive Lung Disease study, the Latin American Project for the Investigation of Obstructive Lung Disease and the European Community Respiratory Health Survey) indicate that a 8.8% reduction of work‐related exposure to noxious gases and dust could prevent a significant proportion of COPD cases, with a 20% decrease of the disease burden.^114^

Knowledge of the principal agents and occupations associated with increased risk of COPD is required in clinical practice to highlight the aetiology of the disease. Knowing the aetiology of COPD could help the clinician in the:

- prevention of a further decline in lung function in patients who are still at work. Occupational exposure to dusts and metals is an independent risk factor for lung function decline^27^;
- identification of a specific and perhaps more troublesome phenotype of COPD; recent studies suggest a role of occupational exposure to VGDF on current disease severity, even in those already retired^115^; and
- compensation of occupational COPD. In some countries, COPD is compensated as an occupational disease when sufficient criteria for causation are satisfied. Moreover, in worker's compensation systems (e.g., Italy), tables with specific exposures/occupation linked to a high probability of COPD are available to guide physicians in reporting occupational disease.

An incomplete list of jobs associated with the occurrence of COPD in UK is provided in Table 1.

**TABLE 1:** List of occupations that may pose at risk of chronic obstructive pulmonary disease in the UK^116^, ^117^

Industry and agriculture	Tertiary sector
Cement workers	Armed forces
Coke oven workers	Building services and sales workers
Construction and trade workers	Building services and sales workers
Farming and agriculture workers	Cleaners
Food products manufacturing	Freight, stock and material handlers
Highway and tunnel workers	Gardeners, park keepers
Inorganic dust‐exposed workers	Healthcare workers
Iron, steel and ferrochrome workers	Records processing and distribution clerks
Mechanic and repair jobs	Repair services/gas station workers
Miners	Sculptors, painters, engravers and art restorer
Paper mill workers	Warehouse stock handlers, stackers
Pottery workers	
Railroad workers	
Rubber, plastics and leather manufacturing workers	
Silicon carbide smelter workers	
Spray painters	
Welders	
Wood workers	

Unfortunately, not all medical programmes worldwide provide sufficient background information on occupational medicine and on work‐related COPD specifically. General practitioners consider their training in occupational medicine to be inadequate.^25^ In fact, occupational medicine education has a very small footprint in most undergraduate medical curricula.^118^ Among postgraduate education, in the United States, only 68.2% of family medicine residency programmes offered specific training in occupational medicine, usually included in the community medicine rotation.^119^ In addition to official educational channels, several government or non‐government agencies offer web‐pages or quick reference guides for health professionals, focused on occupational COPD causes and management (e.g., https://www.hse.gov.uk/copd/causes.htm; https://www.cdc.gov/niosh/programs/resp/risks.html; https://www.safeworkaustralia.gov.au/national‐safe‐work‐month‐2021‐infosheet‐COPD; and https://europeanlung.org/en/information-hub/factsheets/work-related-lung-conditions/).

Knowledge on work‐related COPD is not only limited to those countries with current and frequent exposure to noxious particles and gases, but also to countries where workplace hygiene has improved in recent years, reducing occupational exposures. Occupational COPD has a long latency time between the exposure and diagnosis of the disease,^120^ thus newly diagnosed COPD cases may be caused by exposures which began 20 or more years ago. Furthermore, new risk factors, such as irritants in cleaning agents^83^ and the resurgence of old risk factors in new sectors, such as silica exposure in denim sandblasting^121^ or more recently in artificial stone processing,^122^ could play a role in current and future work‐related COPD cases.

Another important concept to disseminate is the possibility that work‐related exposure could contribute to COPD in ever smokers. As mentioned above, smoking and occupational exposure could interact in starting and maintaining the airway inflammation process, on which COPD is based.^96^

In conclusion, gathering current or past information on workplace exposure is very important for physicians involved in the clinical management of COPD patients. A lack of occupational medicine training is common, but current evidence pushes educational programmes and single physician to fill this gap, also with the support of a specific continuing medical education.^123^

## WHAT A CLINICIAN SHOULD DO?

COPD is a common but often underdiagnosed disease. Clinicians in primary or specialized care should try to facilitate the identification of all missed COPD cases, especially those with a mild disease as the prevention of lung function decline could have a significant effect. This mission is shared with other stakeholders, such as government decision‐makers, national health systems, health insurers, patient support associations and pharmaceutical companies, among others. Unions and employers' association may have a role with their interest in reducing the occupational burden of COPD and the consequences that a severe disease may have on workers' disability and employability. Nearly 40% of patients with COPD must retire prematurely because of the disease^124^ and having COPD can significantly increase the likelihood of absenteeism and presenteeism (Box 2).^125^

**BOX 2:** What a clinician should do

Clinicians should be aware that occupations can contribute to chronic obstructive pulmonary disease (COPD) Can be difficult to retrieve information on current and past occupational exposure, but they can help clinicians to manage better COPD and its socio‐economic consequences There are many online resources that a clinician could use to be informed on this topic and to seek help for her/his patients Depending on local availability, clinician should consider referring the patient to an occupational medicine specialist, occupational health service and/or worker's compensation institution

After the diagnosis, clinicians should collect relevant information on current and past occupational exposures^116^ via an informative occupational history. This is a critical point in primary care^25^ but it is possible to gather important information with few structured questions (see Table 2) on current and past jobs and exposures, focusing on those risk factors (e.g., VGDF) that may be important in COPD pathogenesis. Additional information should be collected on personal protective equipment (PPE) (mainly masks) worn at work and similar symptoms/disease among co‐workers.^126^

**TABLE 2:** Six questions to gather information from occupational history

1. Have you ever been exposed regularly to vapours, gases, dusts and fumes at work?
2. How long have you been exposed?
3. When it was the first time?
4. In which job/jobs?
5. Are you currently exposed?
6. Did you/do you wore/wear personal protective equipment during the exposure?

Exposure duration is an important variable to consider; as with other exposures (e.g., smoking), increasing the exposure duration will increase COPD risk.^90^

Sometimes, it is not easy to have obtain detailed information because:

- the exposure could have been long ago;
- the patient could have no knowledge on the chemical substances used at the workplace;
- the patient could have cognitive problems such as dementia;
- the patient, if still at work, could omit some factors for the fear of losing her/his job or demotion.

In this phase, in agreement with the patient, the clinician could ask for additional information from the occupational health service of the company where the patient is working or, if the patient is retired, from the local occupational health agency responsible for workplace surveillance.

It is not the duty of primary care physicians or specialists in respiratory medicine to find all the criteria required to compensate the worker, but is important to at least raise the suspicion of an occupational contribution.^126^ The process of applying for and having workers' compensation approved will vary considerably between countries. Next steps are referrals to occupational medicine specialists, occupational health services and worker's compensation insurance who will confirm the diagnosis and proceed with other measures (exposure cessation/reduction, compensation, advice, etc.). Raising suspicion in primary care could also help to find COPD clusters or cases related to a peculiar occupational exposure at the local level.

Clinicians should be aware that work‐related COPD can be more complex, and ongoing exposure can cause further lung function decline.^27^ Patients may need clinicians' advice and support in making important decisions on their working life, especially if predisposing factors are present (e.g., AATD). For this reason, it is very important to create a network between the clinicians and the occupational medicine specialists who share care of the same patient.^127^ Nevertheless, since the pioneering studies of Bernardino Ramazzini (1633–1714) in occupational medicine, specialists in this field are also involved in clinical practice, including early diagnosis of work‐related diseases. In this context, health surveillance programmes, targeted to high‐risk groups, can contribute to reduction of the real burden of COPD.^128^ For this reason, statements and guidelines for spirometry standardization and competence assessment are very important in primary care^129^ and occupational medicine.^130^

To sum up, clinicians have a crucial role in reducing COPD underdiagnosis, in raising suspicions of a work‐related contribution for individual patients and in creating a network between clinical and preventive medicine.

## OCCUPATIONAL COPD PREVENTION

Occupational COPD is a preventable disease. There are extensive data on the effect of smoking cessation on COPD prevention,^131^ but little is known on the effect of other preventive measures at the workplace, mainly limited to the effect of PPE.^132^ However, given the existing evidence on the association between COPD and workplace exposure, a preventive intervention plan is reasonable (Box 3).

**BOX 3:** Prevention of occupational COPD

Primary prevention of occupational chronic obstructive pulmonary disease (COPD) is based on risk evaluation, exposure avoidance/reduction, workers' training and education and personal protective equipment use Secondary prevention can be made at primary and specialist (respiratory physicians, occupational medicine specialist) care level towards the definition of at‐risk groups and early diagnosis of occupational COPD Early diagnosis of occupational COPD may help in reducing healthcare and socio‐economic COPD burden Tertiary prevention, made on already diagnosed COPD patient, could help to reduce the severity and the consequences of the diseases

Primary prevention of occupational risk factors involves risk evaluation and then elimination, or if this is not possible, mitigation of the noxious exposure. Risk evaluation is the first and essential step to highlight dangerous exposures and assess the risk at the level of groups and individual workers. Elimination is achieved by substitution of the dangerous substance with another that has no effect on workers' health. If this is not possible (e.g., dust exposure in drilling, mining and tunnel construction), reduction of the exposure to the lowest level reachable is the goal which could be realized by

- engineering controls in the workplace, such as total or partial enclosure with local or general exhaust ventilation (e.g., ventilated booths or rooms), water‐based dust abatement, ventilation and so forth;
- job rotation, good cleaning and maintenance practices and other administrative controls;
- workers' information and training when the worker is assigned to a new task and then continuing;
- correct use of PPE, especially respiratory protective equipment.^116^

While PPE is the final and least protective measure included in the prevention hierarchy, this is the field where most literature on occupational COPD preventive measures is concentrated.^132^, ^133^, ^134^ Primary prevention of occupational COPD should be a significant focus of future research.

Secondary prevention, to intercept the disease in the early stages, is possible through medical surveillance and screening programmes. There are many examples of health surveillance programmes in workers at risk and some attempts have been made to evaluate their cost and efficacy.^135^ Ideally, a medical surveillance programme tailored to the prevention of occupational COPD must include a structured and standardized questionnaire to collect information on respiratory symptoms and their relationship with work. For this purpose, many questionnaires are available; one of the most used is in the European Community Respiratory Health Survey,^136^ but there are others that can consider local factors. One example is available on the webpage of Lung Foundation Australia (https://healthylungsatwork.lungfoundation.com.au/en/quiz).

The questionnaire should be administered at the beginning of employment and periodically; the interval time will depend on exposure level and worker's vulnerability. Information on the questionnaire must be registered periodically and easily accessed to evaluate differences from previous data. Health surveillance must include lung function testing, using the best technical evidence available and specific guidelines on workplace spirometry.^130^

The objective of lung function testing in the workplace is not to diagnose new cases of COPD as in the primary care setting, but to identify workers with a steeper lung function decline, to provide preventive measures to stop or reduce noxious exposures and protect other workers exposed to the same risk factors. A second objective is to target other risk factors which can contribute to the risk of COPD, such as smoking.^137^ Smoking cessation interventions and counselling are already offered worldwide as part of workplace health promotion^138^ and may contribute, together with smoking bans at work, to reducing the burden of COPD. A third objective is to report workers with accelerated decline in lung function to their general practitioner, in order to start a medical surveillance and offer other opportunities to the worker/patient, such as rehabilitation programmes, vaccination, smoking cessation programmes, genetic predisposition screening and, if necessary, pharmacological treatment.

Tertiary prevention is focused on management of the disease in terms of optimizing prevention and reducing health and social consequences. COPD is characterized by reduced quality of life,^1^ medical absence from work, impaired work ability,^139^, ^140^ loss of productivity and indirect cost coming also from short‐term disability.^141^ For these reasons, general practitioners, specialists (in particular respiratory physicians and occupational physicians) and other healthcare professionals must act together to mitigate COPD consequences. This can be pursued by respiratory physician and general practitioners through the best therapeutic strategy, including pulmonary rehabilitation, influenza and pneumonia vaccination. Pneumonia is often a trigger of COPD exacerbations.^1^ Welders exposed to fumes and dusts are at higher risk of pneumonia,^142^ reinforcing the concept of a multifaceted interaction between work and lung health. Worker's compensation is another strategy, if available at the national level, to reduce the socio‐economic burden of occupational COPD.

Other interventions that could help patients to cope with their physical and mental status should be implemented at the primary care and specialist level. In particular, an important role could be played by the occupational physician, who should advise the employer to adapt the patient's occupational tasks and working time to the disease and to its consequences in terms of work ability. In some countries (e.g., Italy), this is a specific duty of occupational physicians involved in workers' health surveillance and it is mandatory for employers to implement their advice.

## CONCLUSIONS

COPD is a multifaceted syndrome caused by noxious particles and gases exposure and characterized by airflow limitation, respiratory symptoms and comorbidities. Smoking is the main risk factor for this disease, but also other risk factors are associated with COPD. Among them, occupational exposure to VGDF plays an important role, defining an ‘occupational’ COPD. Evidence from epidemiological studies support this association, especially for inorganic dust. In population‐based studies, workplace exposure to VGDF accounts for a relevant PAF (14%). The biological plausibility of this association has been demonstrated by several experimental studies.

However, there is still research that needs to be addressed, regarding epidemiological studies, especially cohort studies, on risk factors that seem to increase the risk of COPD, but still without a large evidence base (some irritant substances, some types of organic dust and some types of metal). Furthermore, more epidemiological research on occupational COPD from countries other than North America, Europe and Australia is needed. Moreover, experimental studies exploring the link between new mechanisms leading to COPD (miRNA, DNA methylation and inflammasome activation) and occupational exposures are needed.

From the clinical point of view, occupational COPD has no specific hallmarks compared to COPD in general. This complicates the diagnosis in clinical settings and is responsible for occupational COPD underdiagnosis, together with a lack of education in occupational medicine during medical undergraduate and postgraduate training and education. However, early diagnosis is very important to prevent further decline in lung function due to continuing the exposure and to reduce the health and socio‐economic burden of COPD. For this reason, clinicians should be aware that workplace exposures can be a contributing cause of COPD, even in smokers. Taking an effective occupational history, or just asking a few targeted questions on current and previous workplace exposures, could help to define the occupational burden of COPD at the single patient level in primary and specialist care, where clinicians could propose the first useful interventions (smoking cessation, best therapeutic management, etc.). Afterwards, occupational and public health services will take care of preventive actions (exposure avoidance/reduction, worker's training and heath surveillance, worker's compensation, etc.). A tight collaboration between primary care physicians, respiratory physicians and occupational medicine specialists is desirable and may help to manage COPD patients' health and social issues.

## FUNDING INFORMATION

The study was supported by the University of Perugia. Open Access Funding provided by Universita degli Studi di Perugia within the CRUI‐CARE Agreement.

## CONFLICT OF INTEREST

None declared.

## Supporting information


**Visual Abstract** Occupational COPD: the most under‐recognised occupational lung disease?
