# Supplementary material for: Occupational COPD—The most under‐recognized occupational lung disease?
Source: Respirology. 2022 May 5;27(6):399–410. doi: 10.1111/resp.14272 (PMC9321745; doi:10.1111/resp.14272)
Supplement: Supplementary file 1 — Visual Abstract Occupational COPD: the most under‐recognised occupational lung disease? [file RESP-27-399-s001.pdf]

Occupational COPD
the most under-recognised occupational lung disease?

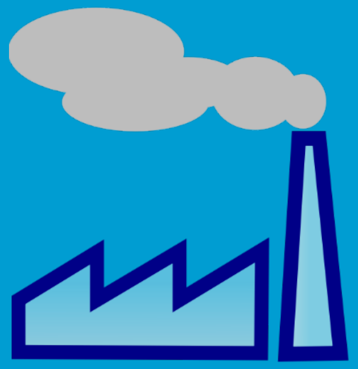

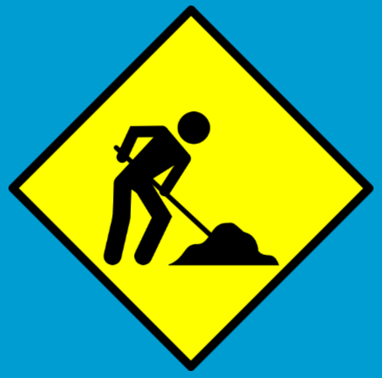

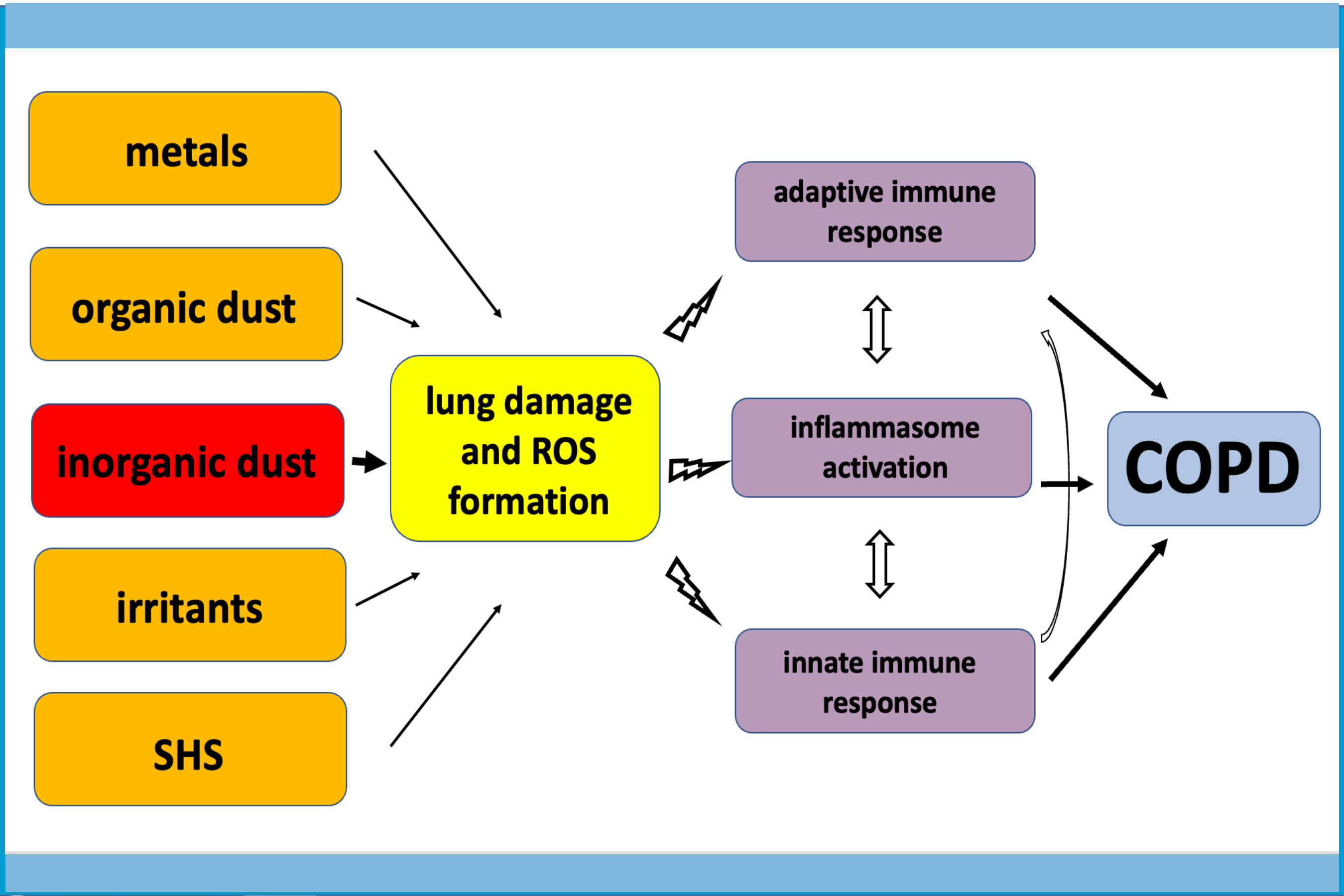

- Occupational COPD is still under-recognised
- Clinicians should be aware that work can contribute significantly to COPD
- COPD can have a substantial impact on work ability
- There is a need of primary, secondary and tertiary prevention
- Clinicians and Occupational Physicians/OHS can collaborate to achieve an effective prevention of COPD

Respirology

Occupational COPD - the most under-recognised occupational lung disease?

Murgia & Gambelunghe, Respirology 2022, https://doi.org/10.1111/resp.14272
